# Association between maternal zinc status and the physical development of infants aged 0–12 months: a cohort study in Northeast China

**DOI:** 10.3389/fnut.2026.1762341

**Published:** 2026-02-20

**Authors:** Juan Dong, Jingru Hou, Shikai Cheng, Linlin Wang, Xuening Li

**Affiliations:** Department of Pediatrics, The Fourth Affiliated Hospital of China Medical University, Huanggu District, Shenyang, China

**Keywords:** cohort, infant, physical development, pregnancy, zinc

## Abstract

**Background:**

Zinc serves as an indispensable element for fetal growth and neonatal development. Presently, the evidence of the relationship between zinc during pregnancy and the physical development (length and weight) of offspring remains inconsistent. The aim of this study was to explore the relationship between them.

**Methods:**

This study recruited pregnant women who gave birth from January 2019 to August 2024. Zinc of these pregnant women in the third trimester pregnancy was tested by atomic absorption spectrometer. Telephone follow-up was carried out when babies were 6 and 12 months old using a structured questionnaire. Multiple linear regressions were used to explore the relationship between maternal zinc status and physical development of infants.

**Results:**

We included a total of 291 mother-child pairs and followed up 249 pairs at 6 months and 216 pairs at 12 months. The maternal zinc deficiency rate was 29.6%. Infants of zinc-sufficient mothers had higher birth weight-for-age *z*-score (β = 0.112; 95% *CI*: 0.004, 0.374) and 6-month weight-for-age *z*-score (β = 0.174; 95% *CI*: 0.113, 0.640) and longer 6-month length-for-age *z*-score (β = 0.165; 95% *CI*: 0.104, 0.699) compared to those of zinc-deficient mothers. In addition, the level of zinc in pregnant women was associated with the weight growth rate (β = 0.120 kg/month; 95% *CI*: 0.001, 0.085) and length growth rate (β = 0.134 cm/month; 95% *CI*: 0.010, 0.221) from 0 to 6 months, but not with the weight-for-length *z*-score at 6 months.

**Conclusions:**

Maternal zinc status is associated with the birth weight and the physical development at 6 months of infants, but adequate zinc does not increase the risk of obesity at 6 months and is not associated with physical development at 12 months.

## Introduction

Children's physical development stands as the paramount indicator for assessing children's growth and health. The theory of the developmental origins of health and disease believes that the nutritional status of children in the uterus and critical pried of growth can affect their lifelong health (1). In the early stage of uman life, pregnancy is a critical period for the development of children. Maternal nutrition during pregnancy plays a pivotal role in fetal growth and development (2). The nutrients consumed by the mother are transferred to the fetus through the placenta, providing the necessary building blocks for fetal growth. Among various nutrients, trace elements are of particular importance due to their involvement in numerous biochemical processes.

Zinc, an essential trace element, has three very basic functions: catalytic, structural, and regulatory, participating in a variety of processes such as DNA synthesis, cell division and growth, immune function, and hormone regulation (3, 4). During pregnancy, the demand for zinc increases to support the rapid growth and development of the fetus. Some studies have shown that zinc deficiency during pregnancy has been associated with adverse pregnancy outcomes, including preterm birth, low birth weight, and impaired fetal growth (5, 6). Presently, there is not entirely consistent on the relationship between maternal zinc level and physical development at birth. A cross-sectional study showed that the serum zinc level of mothers with low birth weight were lower than the serum zinc level of mothers with normal birth weight (7). According to a cohort study based on a large population in China, maternal zinc deficiency during pregnancy elevated the risks of low birth weight and small for gestational age infants (8). Some studies also showed that there was no correlation between maternal zinc concentration and newborn weight and length (9, 10). In addition, most studies have focused solely on physical development at birth, whereas postnatal infant physical development has received comparatively less attention.

Therefore, the aim of this study was to investigate the relationship between maternal zinc status during the third trimester pregnancy and infant weight and length at birth, 6 months and 12 months of age by mother-child pair, to provide a reference basis for the impact of maternal zinc level on early growth and development of children.

## Materials and methods

### Study population

**Figure 1:**
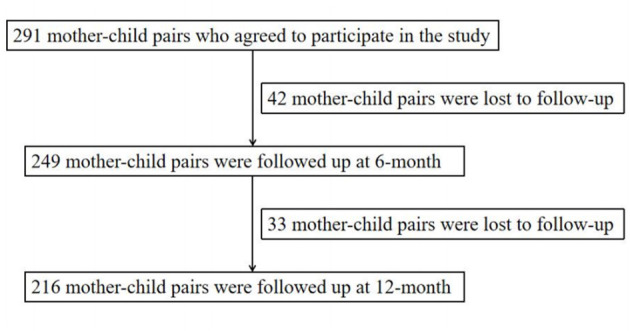
Flow diagram of recruitment and follow-up in this cohort study.

This study was conducted in the Fourth Affiliated Hospital of China Medical University during the period from January 2019 to August 2024. Eligible participants were as follows: (1) pregnant women were over age of 18; (2) single babies with a gestational week of 37–42 weeks. Women with multiple births, birth defects and metabolic diseases were among the exclusion criteria. Data was collected by professional physicians, and a total of 291 mother-child pairs were selected. Telephone follow-up was carried out when babies were 6 and 12 months old. Excluding the babies who were not followed up, a total of 249 mother-child pairs at 6 months and 216 mother-child pairs at 12 months were included in the study. The detailed enrollment process for the study population is shown in Figure 1. Ethical clearance for the study was taken from the ethics committee. All pregnant women have signed the informed consent form.

### Laboratory measurements

We took zinc sampling when mothers were hospitalized for delivery. All participants were required to have fasted for at last 8 h before blood collection, and maternal fasting venous blood was collected in the morning using trace element-specific tubes. Then samples were sent for analysis in the Shenyang Hehe Medical Laboratory. The concentration of zinc in whole blood was quantified using atomic absorption spectrometer. The precision of the method was evaluated using the coefficients of variation (CV). Mean intra-day CV was 4.6% and mean inter-day CV was 4.1%. Maternal zinc level is considered as low if the zinc level is < 76.5 μmol/L, based on the lower limit of the normal reference range established and routinely used by this laboratory. This reference range was derived from the laboratory's internal validation using historical data from the local population and was applied in our hospital's standard clinical practice. It is population-specific rather than an internationally standardized.

### Questionnaires

Each participant had completed the basic information questionnaire for pregnant women, including maternal age, ethnicity, pre-pregnancy weight and late-pregnancy weight. Newborn information such as gender, gravidity, parity, birth weight and birth length was obtained through the birth records. A structured questionnaire was used to collect the feeding patterns and length and weight during the community physical examination of infants aged 6 and 12 months through telephone follow-up. It should be noted that although the relevant data come from the community physical examination, parent-reported anthropometry may still differ from standardized research measurements. The questionnaires have been published in another article (11).

For each pregnant woman, pre-pregnancy body mass index (BMI, kg/m^2^) and weight gain during pregnancy were calculated and categorized in accordance with the Institute of Medicine's recommendations guidelines (12). The weight growth rate is defined as a change in weight over a specific time period expressed as kg/month. The length growth rate is defined as a change in length over a specific time period expressed as cm/month.

### Statistical analysis

Data were analyzed by using SPSS software (v 24.0). Under the assumption that data were lost at random (the loss-to-follow-up analysis of this study supports this assumption), we handled missing data using multiple imputation by chained equation (*m* = 5). The characteristics of pregnant women and infants were summarized using descriptive statistics in this study. Maternal trace element zinc levels were divided into two groups: deficiency and sufficiency. Multiple linear regressions were used to determine the associations between maternal zinc status and physical development of offspring.

We have used the WHO Anthro software to calculate weight-for-age *z*-score, length-for-age *z*-score and weight-for-length *z*-score based on original weight and height data, and used these as outcome variables. The model for birth was adjusted for maternal age, pre-pregnancy BMI (kg/m^2^), maternal weight gain during pregnancy (kg), maternal education level, gender of newborns (male, female), gestational age (weeks), parity (1, ≥2) and cesarean delivery (yes, no). The model for infant physical development at 6 months was adjusted for maternal age, pre-pregnancy BMI (kg/m^2^), maternal weight gain during pregnancy (kg), maternal education level, gender of newborns (male, female), gestational age (weeks), exclusive breast feeding from birth to 6 months (yes, no) and adding supplementary food (yes, no). The model for infants at 12 months was adjusted for maternal age, pre-pregnancy BMI (kg/m^2^), maternal weight gain during pregnancy (kg), maternal education level, gender of newborns (male, female), gestational age (weeks), supplementary food addition (good, average, poor), supplementary food type (diverse, general, single) and outdoor exercise time (< 0.5h, 0.5–1h, >1h). The *p*-value < 0.05 was considered statistically significant. We assessed the normality of model residuals using via Q–Q plots and no meaningful violations were found. Due to the many regression models, we should realize the risk of type-I error inflation. We provided the β and its 95% *CI* at the same time, emphasizing the intensity and accuracy of the association.

## Results

### Population characteristics

A total of 291 mother-child pairs were initially included in the analysis for birth assessment. The number of mother-child pairs with available data decreased to 249 at 6-month follow-up and 216 at 12-month follow-up, because of excluding offspring who refused to follow up. 33.7% of pregnant women were over the age of 30. The majority (81.1%) were of Han ethnicity. The education level of 78.7% of pregnant women was college or above. Cesarean delivery was recorded for nearly half of the women. Maternal and infant characteristics in this study are presented in Table 1.

**Table 1:** Basic characteristics of the study population (*N* = 291).

**Variable classification**		***n* (%) or $\bar{x}$ ±s**
Maternal age (years)	≤ 30	193 (66.3)
	>30	98 (33.7)
Ethnicity	Han	236 (81.1)
	Others	55 (18.9)
Education	High school and less	62 (21.3)
	College or above	229 (78.7)
Pre-pregnancy BMI (kg/m^2^)	< 18.5	45 (15.5)
	18.5–23.9	169 (58.1)
	≥24	77 (26.5)
Gestational weight gain	Inadequate	28 (9.6)
	Adequate	88 (30.2)
	Excessive	175 (60.1)
Gravidity	1	182 (62.5)
	≥2	109 (37.5)
Parity	1	227 (78.0)
	≥2	64 (22.0)
Newborn sex	Male	134 (46.0)
	Female	157 (54.0)
Cesarean delivery	Yes	181 (62.2)
	No	110 (37.8)
Exclusive breastfeeding from birth to 6 months	Yes	128
	No	121
Gestational age (weeks)		38.83 ± 0.91
Birth weight (kg)		3.36 ± 0.37
Birth length (cm)		50.50 ± 1.56
6 months weight (kg)		8.71 ± 1.05
6 months length (cm)		69.39 ± 2.58
12 months weight (kg)		10.47 ± 1.19
12 months length (cm)		77.26 ± 2.79

### Distribution of maternal trace element zinc level

**Figure 2:**
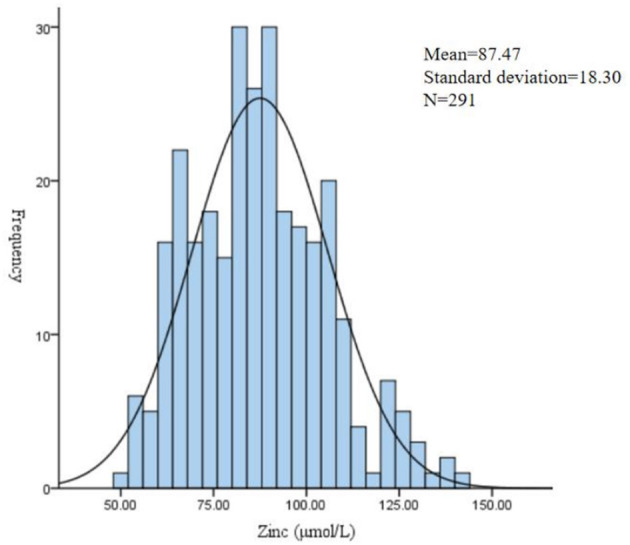
Frequency distribution of trace element zinc level during pregnancy.

The zinc was detected in 291 pregnant women. The median of the zinc was 86.42 μmol/L. The mean of zinc was 87.47 μmol/L, with a standard deviation of 18.30 μmol/L. A total of 29.6% of pregnant women were insufficient in zinc (Table 2 and Figure 2).

**Table 2:** Distribution of trace element zinc among the study population (*N* = 291).

**Zinc status**	***n* (%)**	**$\bar{x}$ ±*s***	**Min**	**25th**	**50th**	**75th**	**Max**
Overall	291	87.47 ± 18.30	50.11	73.52	86.42	99.77	140.09
Deficiency	86 (29.6)	66.49 ± 6.41	50.11	62.66	66.25	71.73	76.28
Sufficiency	205 (70.4)	96.28 ± 13.98	76.51	85.43	92.77	104.73	140.09

### Evaluation of loss-to-follow-up bias

Due to the refusal of follow-up, incomplete information of offspring and other reasons, the follow-up loss rate was 17% at 6 months and 26% at 12 months. To evaluate the loss-to-follow-up bias, we compared whether there was a difference between the baseline group and the maternal zinc status and baseline characteristics of the participants who lost to follow-up at 6 months or 12 months. There was no statistically significant difference (all *p* > 0.05; Supplementary Tables S1 and S2). It is suggested that different zinc status have not led to follow-up loss rate and infants lost to follow-up have similar baseline characteristics. Under the existing data framework, the pattern of loss-to-follow-up is random.

### Associations between maternal zinc status and physical development of infants aged 0–12 months

The average birth weight among neonates born to mothers with sufficient zinc level was 3.38 kg, compared to 3.29 kg for those born to mothers with zinc deficiency. There was a downward trend in the average birth weight in neonates with low zinc level as compared with that with normal zinc level (*p* = 0.057). We did not observe a significant association between the maternal zinc status and birth length. The infant weight at 6 months with maternal low zinc level was observed (8.45 ± 0.85 kg) compared to that of the maternal sufficient zinc level (8.83 ± 1.11 kg), which was statistically highly significant (*p* = 0.003). Similarly, there was a significant correlation between maternal zinc status and length at 6 months (*p* = 0.021). However, maternal zinc status was not associated with infant physical development at 12 months (all *p* > 0.05; Table 3).

**Table 3:** Associations between maternal zinc status and physical development of infants.

**Maternal zinc status**	**Deficiency**	**Sufficiency**	***p***
Birth weight (kg, $\bar{x}\;\pm$*s*)	3.29 ± 0.32	3.38 ± 0.39	0.057
Birth length (cm, $\bar{x}\;\pm$*s*)	50.38 ± 1.55	50.55 ± 1.56	0.418
Weight at 6 months (kg, $\bar{x}\;\pm$*s*)	8.45 ± 0.85	8.83 ± 1.11	0.003
Length at 6 months (cm, $\bar{x}\;\pm$*s*)	68.84 ± 2.42	69.64 ± 2.61	0.021
Weight at 12 months (kg, $\bar{x}\;\pm$*s*)	10.31 ± 0.99	10.54 ± 1.26	0.173
Length at 12 months (cm, $\bar{x}\;\pm$*s*)	76.86 ± 2.54	77.45 ± 2.89	0.150

In the multivariate linear regression analysis, in Table 4, after controlling for confounders, infants of zinc-sufficient mothers had higher birth weight-for-age *z*-score (β = 0.112; 95% *CI*: 0.004, 0.374). However, no correlation between the level of zinc in pregnant women and the length-for-age *z*-score of newborns was observed. In addition, the maternal zinc status showed significantly relationship with the weight-for-age *z*-score (β = 0.174; 95% *CI*: 0.113, 0.640) and length-for-age *z*-score (β = 0.165; 95% *CI*: 0.104, 0.699) of infants at 6 month. However, no significant correlation was found between maternal zinc status and infant physical development at 12 months.

**Table 4:** Associations between maternal zinc status and physical development of infants.

**Characteristics**	**Maternal zinc status**	**Crude**	***p***	**Adjusted**	***p***
		β **(95%** ***CI*****)**		β **(95%** ***CI*****)**	
Birth weight-for-age *z*-score	Deficiency	0		0	
	Sufficiency	0.096 (−0.032, 0.357)	0.101	0.112 (0.004, 0.374)^a^	0.045
Birth length-for-age *z*-score	Deficiency	0		0	
	Sufficiency	0.031 (−0.153, 0.263)	0.601	0.044 (−0.126, 0.285)^a^	0.445
Weight-for-age *z*-score at 6 months	Deficiency	0		0	
	Sufficiency	0.154 (0.064, 0.600)	0.015	0.174 (0.113, 0.640)^b^	0.005
Length-for-age *z*-score at 6 months	Deficiency	0		0	
	Sufficiency	0.122 (−0.006, 0.598)	0.055	0.165 (0.104, 0.699)^b^	0.008
Weight-for-age *z*-score at 12 months	Deficiency	0		0	
	Sufficiency	0.055 (−0.162, 0.387)	0.420	0.036 (−0.206, 0.352)^c^	0.608
Length-for-age *z*-score at 12 months	Deficiency	0		0	
	Sufficiency	0.060 (−0.178, 0.466)	0.379	0.076 (−0.143, 0.507)^c^	0.270

^a^Adjusted for maternal age, pre-pregnancy BMI, maternal weight gain during pregnancy, maternal education level, gender of newborns, gestational age, parity and delivery method. ^b^Adjusted for maternal age, pre-pregnancy BMI, maternal weight gain during pregnancy, maternal education level, gender of newborns, gestational age, exclusive breast feeding from birth to 6 months and adding supplementary food. ^c^Adjusted for maternal age, pre-pregnancy BMI, maternal weight gain during pregnancy, maternal education level, gender of newborns, gestational age, supplementary food addition, supplementary food type, outdoor exercise time.

As shown in Table 5, maternal zinc status was associated with the weight growth rate (β = 0.120 kg/month; 95% *CI*: 0.001, 0.085) and length growth rate (β = 0.134 cm/month; 95% *CI*: 0.010, 0.221) of infants at 6 months, but not associated with the weight and length growth rate of infants at 12 months. In addition, it was also not associated with the weight-for-length *z*-score of infants (Table 6).

**Table 5:** Associations between maternal zinc status and weight and length growth rates of infants.

**Characteristics**		**Maternal zinc status**	**Crude**	***p***	**Adjusted**	***p***
			β **(95%** ***CI*****)**		β **(95%** ***CI*****)**	
0–6 months	Weight growth rates	Deficiency	0		0	
		Sufficiency	0.137 (0.005, 0.092)	0.031	0.120 (0.001, 0.085)^a^	0.047
	Length growth rates	Deficiency	0		0	
		Sufficiency	0.128 (0.004, 0.219)	0.043	0.134 (0.010, 0.221)^a^	0.031
6–12 months	Weight growth rates	Deficiency	0		0	
		Sufficiency	−0.107 (−0.069, 0.008)	0.116	−0.105 (−0.069, 0.009)^b^	0.130
	Length growth rates	Deficiency	0		0	
		Sufficiency	−0.040 (−0.174, 0.094)	0.559	−0.022 (−0.156, 0.112)^b^	0.748

^a^Adjusted for maternal age, pre-pregnancy BMI, maternal weight gain during pregnancy, maternal education level, gender of newborns, gestational age and exclusive breast feeding from birth to 6 months. ^b^Adjusted for maternal age, pre-pregnancy BMI, maternal weight gain during pregnancy, maternal education level, gender of newborns, gestational age, supplementary food addition, supplementary food type, outdoor exercise time.

**Table 6:** Associations between maternal zinc status and weight-for-length of infants.

**Weight-for-length *z*-scores**	**Maternal zinc status**	**Crude**	***p***	**Adjusted**	***p***
		β **(95%** ***CI*****)**		β **(95%** ***CI*****)**	
At 6 months	Deficiency	0		0	
	Sufficiency	0.091 (−0.082, 0.528)	0.152	0.088 (−0.086, 0.518)^a^	0.160
At 12 months	Deficiency	0		0	
	Sufficiency	0.028 (−0.244, 0.369)	0.688	−0.001 (−0.319, 0.313)^b^	0.984

^a^Adjusted for maternal age, pre-pregnancy BMI, maternal weight gain during pregnancy, maternal education level, gender of newborns, gestational age and exclusive breast feeding from birth to 6 months. ^b^Adjusted for maternal age, pre-pregnancy BMI, maternal weight gain during pregnancy, maternal education level, gender of newborns, gestational age, supplementary food addition, supplementary food type, outdoor exercise time.

## Discussion

We have revealed a dynamic association between maternal zinc status and infant physical development during the first year of life. We observed that the zinc status of pregnant women was associated with birth weight and both weight and length at 6 months, without increasing the obesity rate at 6 months. Therefore, the critical role of maternal zinc nutrition in shaping the early growth trajectory of children is worth paying attention to. Low birth weight and childhood stunting remain critical public health challenges. Ensuring adequate zinc nutrition during pregnancy is a crucial defense.

Among 291 pregnant women, 29.6% experienced zinc deficiency, which was consistent with findings of Iran's Second National Micronutrient Survey (28.0%) (13). This may be significantly linked to the dietary patterns in Northeast China. Research showed that animal-derived zinc was a good source of dietary zinc (14). The diet in this region is mainly grain beans, which leads to insufficient intake of zinc.

In this study, we have found that adequate maternal zinc level during pregnancy was associated with higher birth weight-for-age *z*-score. This was in line with several previous studies (7, 15). However, it is important to note that the observed effect size, such as a β-coefficient of approximately 0.112 for birth weight-for-age *z*-score, is modest in absolute terms. At the individual level, a difference of this magnitude at the individual level may not be clinically decisive for a healthy term infant. However, at the population level, even a small increase in the average birth weight-for-age *z*-score may be translated into a considerable reduction in the incidence of low birth weight. From a biological perspective, zinc is an essential trace element for the synthesis of DNA and RNA, and directly participates in the replication of genetic material and cell division process (2, 16). Zinc deficiency during pregnancy directly affects the supply of zinc to the fetus, with the most immediate consequences being intrauterine growth restriction and low birth weight. In addition, the placenta as one of the important appendages of the fetus, is the basis for the normal growth of the fetus and zinc is involved in many functions of the placenta (endocrine, metabolic, and development) (17). In contrast, the lack of association with birth length may reflect the difference of linear growth in utero. The growth of bones is a relatively slow process, which may be less sensitive to short-term fluctuations of trace elements during pregnancy (18).

Until now, there are few reports analyzing the long-term impact of zinc level during pregnancy on infant physical development. In this study, we continued to track the physical development of infants. The most compelling finding of our study was the significant association of maternal zinc status with physical development of infants at 6 months. We propose some possible mechanisms to explain this phenomenon. First, zinc is involved in the regulation of metabolic processes and hormone secretion in both the mother and the fetus. It can influence insulin sensitivity and the secretion of growth—related hormones, such as insulin—like growth factor–1 (19, 20). These hormones play a crucial role in promoting fetal growth and postnatal physical development. Second, the transport of zinc to the fetus is fully dependent on its concentration in the mother's blood (16). Mother's sufficient zinc provides higher zinc reserves for the offspring, so that they have higher growth potential after birth. Third, zinc's crucial role in immune function cannot be overlooked (21, 22). Adequate zinc status can enhance immune competence and reduce the incidence of infections, and thereby reduce infection-mediated growth faltering during this vulnerable window around 6 months of age.

Early childhood growth trajectories and rapid catch-up growth have been recognized as potential risk factors for future metabolic syndromes, such as obesity, hypertension and diabetes (23). This has raised our concern about the possible long-term metabolic risks that may accompany the above-mentioned growth-promoting benefits. In order to preliminarily assess this risk, we analyzed the obesity of 6-month-old infants in this cohort study. The results showed that the obesity rate of this population was 6.4%, which is basically equivalent to the current data (24). Moreover, maternal zinc status was not associated with weight-for-length *z*-score of infants at 6 months. Therefore, although the zinc-sufficient group showed a faster early growth trend, it did not lead to a significant increase in obesity at the age of 6 months.

We also continued to track the physical development of offspring at 12 months. We observed that maternal zinc status was not associated with the physical development of infants at 12 months and the weight and length growth rate of 6–12 months. As kids grow older, their growth trajectories begin to converge toward their genetic potential (25). In addition, the transformation of nutrition pattern is also a key factor. At the age of 6–12 months, infants experience an important transition from pure breast milk or formula feeding to diversified complementary foods. The intake of other nutrients has a direct and powerful driving effect on physical development, which may partially cover up the long-term effect of the prenatal single micronutrient status. Meanwhile, the influence of environmental and socioeconomic factors is increasingly prominent. The growth and development of infants are more susceptible to the combined influence of multiple complex factors such as family feeding behavior, hygiene conditions and the broader socioeconomic environment. In the future, we will continue to carry out prospective follow-up visits to further explore the long-term effects of zinc nutrition during pregnancy on the health of children.

Our study had some limitations. First, the sample size was relatively small and this was a single-center study. So the results may not apply to populations in other regions. Second, maternal zinc concentration was measured only at a single time point in the third trimester, which may not accurately represent zinc exposure earlier in pregnancy when organogenesis and placental development are more influenced. Therefore, the associations observed in this study may primarily reflect the relationship between late-pregnancy zinc status and infant physical development. The single-time-point measurement design limits our ability to establish causal inferences between zinc exposure at different gestational stages and offspring development, and also restricts temporal interpretation regarding critical windows of zinc influence. In addition, maternal zinc is not fully equivalent to or accurately reflects the actual zinc level of fetus and we study did not measure the neonatal zinc concentrations, which limits the possible mechanism explanation of our study founding. Third, the zinc critical value we use is the lower limit of the reference value established by our laboratory based on the local population, which is used to classify the zinc nutrition status of pregnant women in the context of this study. Although this ensures consistency with the clinical context of this study, it may be limited to direct comparison with other studies using different standards. Fourth, our growth assessment was limited to somatic parameters (weight and length) and did not include the measurement of head circumference at birth or during follow-up. Head circumference is a key indicator of brain growth, and zinc plays an indispensable role in neurodevelopment. This omission weaken the biological integrity and the depth of explanation of the developmental significance found in this study. Fifth, the evaluation of maternal nutrition was limited to BMI and gestational weight gain and failed to collect quantitative dietary intake data. Therefore, the observed association may reflect a broader pattern of maternal undernutrition rather than the specific effect of zinc. This constitutes an important source of residual mixing. Additionally, there are many factors affecting the physical development of infants aged 0–12 months and we have not accounted for all potential confounding factors, such as maternal dietary intake, zinc supplement use, socioeconomic status, maternal stress and exposure to environmental toxins. The results may overestimate the independent role of the maternal zinc. Finally, this study only analyzed the effects of maternal zinc deficiency during pregnancy on infants aged 0–12 months. Actually, some studies have shown that maternal deficiency of other nutrients, such as iron and folic acid, increased the risks of low birth weight (26, 27). And in the pregnant population, zinc deficiency often coexists with other nutrients. Since we have not measured the biomarkers of these related nutrients simultaneously. we cannot adjust their potential mixed effects or conduct sensitivity analysis to separate the specific contribution of zinc. Therefore, the observed association may not the independence of zinc alone. The result should be interpreted with caution. In the future, large-scale, multi-center, prospective studies are needed to find out the validity of our findings. Meanwhile, such studies should not only be limited to the anthropometric measures, but also incorporate longitudinal neurodevelopmental outcomes to more comprehensively evaluate the impact of maternal zinc on the development of offspring.

## Conclusions

In this study, we found that the impact of maternal zinc on offspring growth was not static but evolved over the first year, with a pronounced effect at the critical 6-month juncture. This suggests that adequate zinc level during pregnancy may play a significant role in early infant growth. We should increase attention to maternal nutrition, specifically zinc intake, to promote growth and development in the next generation. Future studies should further explore the long-term health consequences of prenatal zinc deficiency, including physical and neurological development and investigate the effective interventions to reduce its adverse effects.

## Data Availability

The original contributions presented in the study are included in the article/Supplementary material, further inquiries can be directed to the corresponding author.

## References

[1] Hoffman DJ Reynolds RM Hardy DB. Developmental origins of health and disease: current knowledge and potential mechanisms. Nutr Rev. (2017) 75:951–70. doi: 10.1093/nutrit/nux053 29186623

[2] Young MF Ramakrishnan U. Maternal undernutrition before and during pregnancy and offspring health and development. Ann Nutr Metab. (2021) 1–13. doi: 10.1159/000510595 33524980

[3] King JC. Zinc: an essential but elusive nutrient. Am J Clin Nutr. (2011) 94:679S−84S. doi: 10.3945/ajcn.110.005744 21715515 PMC3142737

[4] Terrin G Berni Canani R Di Chiara M Pietravalle A Aleandri V Conte F . Zinc in early life: a key element in the fetus and preterm neonate. Nutrients. (2015) 7:10427–46. doi: 10.3390/nu7125542 26690476 PMC4690094

[5] Kumari D Garg S Bhawrani P. Zinc homeostasis in immunity and its association with preterm births. Scand J Immunol. (2022) 95:e13142. doi: 10.1111/sji.13142 35007353

[6] Scheplyagina LA. Impact of the mother's zinc deficiency on the woman's and newborn's health status. J Trace Elem Med Biol. (2005) 19:29–35. doi: 10.1016/j.jtemb.2005.07.008 16240669

[7] Karim S Fazal S Naeem M Ali H Fazal R Karim A . Frequency of low birth weight and its relationship with maternal serum zinc: a cross-sectional study. Ann Med Surg (Lond). (2023) 85:2469–73. doi: 10.1097/MS9.0000000000000687 37363444 PMC10289759

[8] Wang H Hu YF Hao JH Chen YH Su PY Wang Y . Maternal zinc deficiency during pregnancy elevates the risks of fetal growth restriction: a population-based birth cohort study. Sci Rep. (2015) 5:11262. doi: 10.1038/srep11262 26053136 PMC4459238

[9] Samimi M Asemi Z Taghizadeh M Azarbad Z Rahimi-Foroushani A Sarahroodi S. Concentrations of serum zinc, hemoglobin and ferritin among pregnant women and their effects on birth outcomes in Kashan, Iran. Oman Med J. (2012) 27:40–5. doi: 10.5001/omj.2012.08 22359724 PMC3282140

[10] Daniali SS Yazdi M Heidari-Beni M Taheri E Zarean E Goli P . Birth size outcomes in relation to maternal blood levels of some essential and toxic elements. Biol Trace Elem Res. (2023) 201:4–13. doi: 10.1007/s12011-022-03121-w 35298828

[11] Li X Chen Q Wu D Xiao Z Shi C Dong Y . High levels of BPA and BPF exposure during pregnancy are associated with lower birth weight in Shenyang in Northeast China. Chem Res Toxicol. (2024) 37:1199–209. doi: 10.1021/acs.chemrestox.4c00145 38953537

[12] Goldstein RF Abell SK Ranasinha S Misso M Boyle JA Black MH . Association of gestational weight gain with maternal and infant outcomes: a systematic review and meta-analysis. JAMA. (2017) 317:2207–25. doi: 10.1001/jama.2017.3635 28586887 PMC5815056

[13] Pouraram H Djazayery A Mohammad K Parsaeian M Abdollahi Z Dorosty Motlagh A . Second national integrated micronutrient survey in Iran: study design and preliminary findings. Arch Iran Med. (2018) 21:137–44. 29693403

[14] Lee YA Hwang JY Kim H Ha EH Park H Ha M . Relationships of maternal zinc intake from animal foods with fetal growth. Br J Nutr. (2011) 106:237–42. doi: 10.1017/S0007114510005878 21338540

[15] Alemu B Gashu D. Association of maternal anthropometry, hemoglobin and serum zinc concentration during pregnancy with birth weight. Early Hum Dev. (2020) 142:104949. doi: 10.1016/j.earlhumdev.2019.104949 31923646

[16] Grzeszczak K Kwiatkowski S Kosik-Bogacka D. The role of Fe, Zn, and Cu in pregnancy. Biomolecules. (2020) 10:1176. doi: 10.3390/biom10081176 32806787 PMC7463674

[17] Garner TB Hester JM Carothers A Diaz FJ. Role of zinc in female reproduction. Biol Reprod. (2021) 104:976–94. doi: 10.1093/biolre/ioab023 33598687 PMC8599883

[18] Ohuma EO Villar J Feng Y Xiao L Salomon L Barros FC . Fetal growth velocity standards from the fetal growth longitudinal study of the INTERGROWTH-21st project. Am J Obstet Gynecol. (2021) 224:208.e1–18. doi: 10.1016/j.ajog.2020.07.054 PMC7858163 32768431

[19] McNall AD Etherton TD Fosmire GJ. The impaired growth induced by zinc deficiency in rats is associated with decreased expression of the hepatic insulin-like growth factor I and growth hormone receptor genes. J Nutr. (1995) 125:874–9. doi: 10.1093/jn/125.4.874 7722689

[20] Miletta MC Schöni MH Kernland K Mullis PE Petkovic V. The role of zinc dynamics in growth hormone secretion. Horm Res Paediatr. (2013) 80:381–9. doi: 10.1159/000355408 24296719

[21] Wessels I Maywald M Rink L. Zinc as a gatekeeper of immune function. Nutrients. (2017) 9:1286. doi: 10.3390/nu9121286 29186856 PMC5748737

[22] Hojyo S Fukada T. Roles of zinc signaling in the immune system. J Immunol Res. (2016) 2016:6762343. doi: 10.1155/2016/6762343 27872866 PMC5107842

[23] Drozdz D Alvarez-Pitti J Wójcik M Borghi C Gabbianelli R Mazur A . Obesity and cardiometabolic risk factors: from childhood to adulthood. Nutrients. (2021) 13:4176. doi: 10.3390/nu13114176 34836431 PMC8624977

[24] Guo Y Yin X Wu H Chai X Yang X. Trends in overweight and obesity among children and adolescents in China from 1991 to 2015: a meta-analysis. Int J Environ Res Public Health. (2019) 16:4656. doi: 10.3390/ijerph16234656 31766709 PMC6926698

[25] Livshits G Peter I Vainder M Hauspie R. Genetic analysis of growth curve parameters of body weight, height and head circumference. Ann Hum Biol. (2000) 27:299–312. doi: 10.1080/030144600282181 10834294

[26] Xiong J Zhou W Huang S Xu K Xu Y He X. Maternal anaemia and birth weight: a cross-sectional study from Jiangxi Province, China. Fam Pract. (2023) 40:722–7. doi: 10.1093/fampra/cmac148 36610700

[27] Sámano R Martínez-Rojano H Chico-Barba G Gamboa R Tolentino M Toledo-Barrera AX . Serum folate, red blood cell folate, and zinc serum levels are related with gestational weight gain and offspring's birth-weight of adolescent mothers. Nutrients. (2024) 16:1632. doi: 10.3390/nu16111632 38892565 PMC11174574

