# Supplementary material for: Association between maternal zinc status and the physical development of infants aged 0–12 months: a cohort study in Northeast China
Source: Front Nutr. 2026 Feb 20;13:1762341. doi: 10.3389/fnut.2026.1762341 (PMC12963010; doi:10.3389/fnut.2026.1762341)
Supplement: Supplementary file 1 [file Table_1.docx]

**Supplementary Table S1** Comparison of baseline characteristics between initial participants and those lost to follow-up at 6 months

| baseline characteristics |  | initial participants | loss-to-follow-up at 6 months | *P* |
| --- | --- | --- | --- | --- |
| maternal zinc status | deficiency | 86 (29.6) | 8 (19.0) | 0.157 |
|  | sufficiency | 205 (70.4) | 34 (81.0) |  |
| maternal age (years) | ≤30 | 193 (66.3) | 27 (64.3) | 0.794 |
|  | ＞30 | 98 (33.7) | 15 (35.7) |  |
| ethnicity | Han | 236 (81.1) | 33 (78.6) | 0.697 |
|  | others | 55 (18.9) | 9 (21.4) |  |
| education | High school and less | 62 (21.3) | 8 (19.0) | 0.737 |
|  | College or above | 229 (78.7) | 34 (81.0) |  |
| pre-pregnancy BMI (kg/m^2^) | ＜18.5 | 45 (15.5) | 7 (16.7) | 0.587 |
|  | 18.5-23.9 | 169 (58.1) | 27 (64.3) |  |
|  | ≥24 | 77 (26.5) | 8 (19.0) |  |
| gestational weight gain | inadequate | 28 (9.6) | 1 (2.4) | 0.257 |
|  | adequate | 88 (30.2) | 12 (28.6) |  |
|  | excessive | 175 (60.1) | 29 (69.0) |  |
| gravidity | 1 | 182 (62.5) | 20 (47.6) | 0.064 |
|  | ≥2 | 109 (37.5) | 22 (52.4) |  |
| parity | 1 | 227 (78.0) | 31 (73.8) | 0.543 |
|  | ≥2 | 64 (22.0) | 11 (26.2) |  |
| newborn sex | male | 134 (46.0) | 19 (45.2) | 0.922 |
|  | female | 157 (54.0) | 23 (54.8) |  |
| cesarean delivery | yes | 181 (62.2) | 24 (57.1) | 0.592 |
|  | no | 110 (37.8) | 18 (42.9) |  |
| gestational age (weeks) |  | 38.83±0.91 | 39.12±0.77 | 0.053 |
| birth weight (kg) |  | 3.36±0.37 | 3.34±0.35 | 0.769 |
| birth length (cm) |  | 50.50±1.56 | 50.52±1.63 | 0.921 |

**Supplementary Table S2** Comparison of baseline characteristics between initial participants and those lost to follow-up at 12 months

| baseline characteristics |  | initial participants | loss-to-follow-up at 12 months | *P* |
| --- | --- | --- | --- | --- |
| maternal zinc status | deficiency | 86 (29.6) | 16 (21.3) | 0.157 |
|  | sufficiency | 205 (70.4) | 59 (78.7) |  |
| maternal age (years) | ≤30 | 193 (66.3) | 47 (62.7) | 0.552 |
|  | ＞30 | 98 (33.7) | 28 (37.3) |  |
| ethnicity | Han | 236 (81.1) | 59 (78.7) | 0.635 |
|  | others | 55 (18.9) | 16 (21.3) |  |
| education | High school and less | 62 (21.3) | 18 (24.0) | 0.615 |
|  | College or above | 229 (78.7) | 57 (76.0) |  |
| pre-pregnancy BMI (kg/m^2^) | ＜18.5 | 45 (15.5) | 11 (14.7) | 0.955 |
|  | 18.5-23.9 | 169 (58.1) | 45 (60.0) |  |
|  | ≥24 | 77 (26.5) | 19 (25.3) |  |
| gestational weight gain | inadequate | 28 (9.6) | 6 (8.0) | 0.911 |
|  | adequate | 88 (30.2) | 23 (30.7) |  |
|  | excessive | 175 (60.1) | 46 (61.3) |  |
| gravidity | 1 | 182 (62.5) | 41 (54.7) | 0.213 |
|  | ≥2 | 109 (37.5) | 34 (45.3) |  |
| parity | 1 | 227 (78.0) | 55 (73.3) | 0.391 |
|  | ≥2 | 64 (22.0) | 20 (26.7) |  |
| newborn sex | male | 134 (46.0) | 34 (45.3) | 0.912 |
|  | female | 157 (54.0) | 41 (54.7) |  |
| cesarean delivery | yes | 181 (62.2) | 43 (57.3) | 0.441 |
|  | no | 110 (37.8) | 32 (42.7) |  |
| gestational age (weeks) |  | 38.83±0.91 | 38.99±0.85 | 0.183 |
| birth weight (kg) |  | 3.36±0.37 | 3.35±0.35 | 0.941 |
| birth length (cm) |  | 50.50±1.56 | 50.47±1.48 | 0.874 |
